# Notes on Medical Matters and Medical Men in Paris

**Published:** 1847-07

**Authors:** David W. Yandell

**Affiliations:** Louisville, Ky.; Paris


**Art. II.**—*Notes on Medical Matters and Medical Men in Paris.* By David W. Yandell, M.D., of Louisville, Ky.

I should, according to promise, proceed now to give an account of the use of sulphuric ether by the Paris obstetricians in cases of difficult labor, but so much has already found its way into the journals on this subject that I have hesitated about fulfilling my promise. I am the less inclined to add any thing at present to the numerous details published, because I perceive that some of our own practitioners have made application of the new agent in such cases, and suppose that before this letter can reach you the subject will have grown old in America. Before now, it may be presumed, trials have been made with it by many of our own physicians, and I should not wonder if reports of its successful employment were to go forth with this letter from some of your correspondents. Leaving the subject, therefore, for the present, I

go on with my usual report of such interesting novelties as I see in the hospitals, and meet with in the European journals of medicine.

*Ergotine in external hemorrhage.*—At the meeting of the Academy of Sciences of the 22d of March, a letter was read from M. Bonjean, a physician at Chambourg, giving an account of some successful experiments with ergotine in external hemorrhage. He had made a previous communication on the same subject, I believe. He mentioned a case in which the radial artery had been severed, hemorrhage from which was arrested without ligature; and he also stated that ergotine had been successfully used in the treatment of scurvy.

*Solvent for stone in the bladder.*—The secretary of the Academy of Naples, M. Ferdinand de Luca, wrote a letter to the academy, in which he states that M. Cervelleri, a surgeon of the military hospital of Naples, has succeeded in dissolving stone in the bladder of living persons, by the use of electricity. He mentioned, however, only one case, from which, of course, one cannot pretend to appreciate the merits of the experiment. The patient in this case was a female, and the stone being placed in contact with the wires from a voltaic battery, was reduced in half an hour, and without pain, to fragments sufficiently small to be voided in the natural way.

M. de Luca alludes to several other cases, and as the question is one of such importance, I regret that he did not enter into those details and advance that proof which science requires.

*Ether in its effects on the blood and in pregnancy.*—At the sitting of the Academy of March 8th, M. Roux communicated the results of some experiments with the inhalation of ether on patients. In some cases, he states, the patients lose the equilibrium of motion without the slightest disturbance of the intellect, and that the suffocation from ether differs from all others, and particularly from that caused by carbonic acid, in which the blood, completely deprived of its oxygen, is black, whereas with all persons whom he had etherized, the arterial and venous blood retained the natural color. The blood, however, according to Messsrs. Lenoir and Voillemier, and many other surgeons, has greater fluidity than when in a normal state.

M. Jacques Cardan gave an account of some serious results from the administration of ether to females in a state of pregnancy. He considers it highly improper to etherize women when in this condition.

*A new sign of real death.*—M. Bouchut made a statement relative to the pretended discovery of the means of dstinguishing real from apparent death by burning. It has been asserted, by some one in Germany, if I am not mistaken, that if life be in the body, a burn of the second degree will cause a blister to rise, whereas no blister can be raised by this means upon a corpse. M. Bouchut states that he tried the experiment upon four dead bodies, and produced blisters precisely similar to what would have been raised by the same means on the living subject. Bouchut but confirms the experiments already made, and with similar results, by several other surgeons. He proposes, as the best means of ascertaining whether life be extinct or not, a prolonged auscultation of the region of the heart; but he does not pretend that this alone is conclusive.

*Nervous fluid and electricity.*—At a meeting of the Academy of Sciences, March 15th, M. Dumas communicated a note from M. Matteuci on the nervous fluid and the influence of electricity. His conclusions are as follows:

1. The nervous fluid is produced by the chemical action of nutrition.

2. It is principally developed in the muscles, and, possessed of a repulsive force between its parts, like the electric fluid, keeps the elements of the muscular fibre in a state of repulsion similar to that presented by electrified bodies.

3. When the nervous fluid ceases to be free in the muscle, the elements of the muscular fibre are attracted to each other,

4. The nervous fluid constantly penetrates into the nerves and thence to the brain, assuming in these parts a new state, which is no longer that of a free fluid. According to the quantity of the fluid which ceases to be free in the muscle, the contraction is more or less strong.

5. This state is that of the nervous current, or the kind of discharge, which proceeds from the nervous extremities to the brain, and returns in a contrary state by the action of the will.

6. When this discharge takes place, the muscular contraction ensues, the fluid ceasing to be free in the muscles.

7. This discharge of the nervous fluid, acting as in electrical fishes, explains the contraction in both cases, and by the same arrangement of the parts the nervous current produces an electrical polarization.

8. The electric current prevents the nervous discharge if it be directed in the same state, which is the direct current: the nervous fluid not being able to enter and concentrate itself in the nerve, the latter loses its excitability; the contrary ensues for the opposite current, by which the nervous fluid accumulates in the nerve and augments its sensibility.

*Ether injected into the b'oodvessels.*—I forgot to mention that at the sitting of March 22d, M. Flourens communicated the result of some experiments as to the action of ether taken internally and injected into the arteries. He administered to a number of dogs sulphuric ether in doses varying from six to twenty-four grammes. All of these animals, says M. Flourens, suffered severely, and some of them died; others were intoxicated, but not one was etherized, that is, struck with general and total insensibility, which is the characteristic condition of etherization. Neither, says M. F., did the injection of ether into the arteries produce etherization, but it produced what he termed "a phenomenon:" when an animal is subjected to the ethereal inhalation or ingestion, the spinal marrow loses the principle of feeling before it does that of motion. This is not the case when ether is injected into an artery; motion then ceases before insensibility to pain commences.

*Acupuncture in aneurism.*—The Scientific Congress of Genoa having appointed a committee, in September last, to examine the effects of electro-puncture employed as a means for coagulating the blood in the arteries, and causing their obliteration, Dr. Arson, the chairman of this committee, has made a report, of which the following are the principal conclusions:

1. By means of the electro-puncture the blood may be coagulated in the bloodvessels in such a way that there will result a solid fibrinous mass, which adheres to the walls of the vessel, and which completely intercepts the circulation of the blood.

2. At the end of ten, twenty and sometimes thirty minutes, the mass is sufficiently solidified to obstruct the vessel.

3. The same phenomenon occurs in the veins as in the arteries, with this difference, that the solidified mass obtained in the veins is a little less consistent and more deeply colored than that of the arteries.

4. The mass or coagulum which results from the double ligature, and which is found compressed, is less colored than that which is produced by the galvano-puncture.

5. The solid mass comprised between two ligatures and submitted to the electric current during life, presents the same characters as that which is formed between two ligatures without any electrical current; which proves the necessity of leaving the part submitted to the experiment under the influence of the circulation.

6. The blood drawn from the vessels and submitted to the electric current does not coagulate, contrary to the opinion expressed by Dr. Pétrequin.

7. The coagula may be produced without cauterizing the arterial tissue, and without giving rise to any serious disorders in the economy, it being sufficient merely to keep up a constant electric current.

8. The hemorrhage which appears after the extraction of the needles which have served as the conductors of the electricity, is insignificant and easily arrested by the simple application of cold water.

*New mode of applying electricity.*—Prof. Orioli called the attention of the congress to the happy results obtained by Dr. Cogeina and himself in the following application of electricity: Unite a zinc and silver plate by means of a silver wire. Having now made two little wounds on the portion of the cutaneous surface that you desire to act upon about four lines in diameter, with a view of giving to the skin a better conducting power, which you may do with any epispastic that you deem preferable, and before any other alteration of the skin takes place, applying to the denuded surface the two metallic plates, it will be observed that the one which corresponds to the zinc will enlarge, become more profound and give place to a sort of eschar; assuming first a white and then a black color. Applied in this manner, the apparatus may remain for a number of hours, but not longer than a day or two, for fear of carrying the irritation of the part too far.

The authors see in this procedure a double advantage: 1st. A powerful irritation analogous to that produced by epispastics, and superior to that produced by moxas and other similar means—an irritation by which we may overcome as by enchantment certain very obstinate and rebellious affections. Among other examples, they cite the recovery of a young girl who for five years had been tormented with an almost continual cough. 2d. A very active agent to change the surface of unhealthy ulcers, and an emunctory by which very distressing wounds, that were disposed to become carcinomatous, are perfectly cicatrized.

*Test for water in alcohol.*—In no country are physicians more interested in knowing whether the alcohol they use is free from water than in the United States. There are various methods known to almost every one for ascertaining this, but there seems to me none so simple, and few, I am inclined to think, which succeed so well as that of M. Casoria, published in the Journal of Medical Chemistry. It is based upon the property possessed by the common hydrated sulphate of copper of losing its color when it becomes dry, and regaining it when again brought in contact with water. Thus, if we place a piece of anhydrous sulphate of copper in a vessel containing the alcohol which it is wished to test, in a short time it becomes blue if the alcohol be mixed with water, whereas if it is absolute the salt will remain white.

*Opium in emphysema.*—M. Louis is in the habit of employing opium in very large doses in the treatment of emphysematous attacks. I use this rather doubtful expression because I would not have you for a moment to suppose, that M. Louis pretends to cure by opium the physical alterations which characterize vesicular emphysema of the lung.

In emphysema, as in the greater part of those affections which offer an intermittent or remittent type, there are two indications for the practitioner to fulfil when called to the bed side of the patient: first, to combat the actual attack, the dyspnœa, the cough, the threatening suffocation of the emphysematous; then the disease itself, of which these phenomena are but the symptoms or consequence. As it is impossible to overcome the material organic lesions in emphysema, it is his duty to direct his efforts towards the palliation of the affection. A short time since there was a well characterized case of emphysema of the lungs in a woman fifty-seven years of age in the wards of M. Louis. There was exaggerated sonorousness of the chest on percussion; almost complete absence of the respiratory murmur; souflant and sibilant rales. The patient had been exceedingly subject to asthmatic attacks, for which she was in the habit of being bled, although it afforded her no particular relief. M. Louis, without absolutely proscribing bloodletting in such cases, thinks that its employment should be the exception rather than the rule; and contented himself with prescribing the following potion: Gum julep iv ℥, laudanum of Sydenham xx gtt., hydrochlorate of morphia 1-5 gr.

On the next day a notable amelioration was observed in the condition of the patient, and has persisted under the influence of the preparations of opium, which have been continued, though in smaller doses.

M. Louis remarked that this case reminded him of one that he saw some twelve years ago, in which the action of the opium was more rapid than he had ever seen it. He was called one day to a young lady seventeen or eighteen years of age, who was laboring under an attack of suffocation. At first he supposed she had some organic affection of the heart, though percussion and auscultation soon revealed to him the true nature of the disease, which was emphysema. He immediately prescribed for the young patient two grains of opium, and at the end of a couple of hours she was relieved.

*Hydatids of the spinal cord.*—The following case is another added to the very small number on record, of hydatids of the spinal cord. Occurring in one of the wards in which I take one of my private courses on medical diagnosis, and having daily witnessed the train of symptoms to which these morbid products gave rise, besides being present at the autopsy, you will bear with my somewhat detailed account of the case. The patient was a man aged twenty-five years, who exercised the calling of sockmaker. His father died at the advanced age of seventy-four years, after having enjoyed uninterruptedly good health. His mother, whose health is excellent, is in her sixty-third year; she has given birth to thirteen children, five of whom died young, though the patient did not know of what disease; the others are in good health. The health of the patient himself was good during his childhood. In his twelfth year he had an attack of measles, soon after which supervened a blepharotis that exists at this time.

It is now nine years since he came to Paris, and eighteen months since he received a blow on the back, which gave him considerable pain, and produced, he said, a momentary embarrassment of his respiration. The following days he felt a pain in the dorsal region, which at times became very severe, extending to the chest, where it produced a burning sensation. He now consulted a physician, who found the pain was augmented by pressure on the injured point of the spine, to which he ordered sixteen leeches; after which the pain lost its acuteness, though occasionally a dull, vague pain was felt in the back, especially after fatigue.

Four months ago, having resumed his occupation, the acute pain reappeared at the same spot, and has seemed daily to increase, being occasionally accompanied by a burning sensation in the back, and a feeling of constriction in the walls of the thorax while walking. In a few days he was obliged to relinquish his work and enter a hospital, at which period he suffered a good deal when standing up or lying on either side, though when on his back or when he bent backwards he experienced no pain. At the hospital twenty-five leeches were applied to the dorsal region, and afterwards a blister that was kept open for twelve days, during which time the pain disappeared; but as soon as the blister was allowed to dry up, dull pains supervened in the walls of the chest, which, however, yielded to cups applied to their seat.

After the lapse of twenty-one days, the patient left the hospital, being decidedly relieved, but still feeling the dull pain in the back. This in the course of a couple of weeks again became acute, and, accompanied by the constriction of the chest, reappeared from time to time until the first of January, when he felt his legs become feeble. It seemed to him that he danced while walking. The movements of his legs were not in concert, and he was in continual fear that he should fall on the persons who passed him. Towards the end of January the patient felt a pain in the abdomen as if he had been struck there, from which moment the painful condition of the chest to which I have alluded disappeared; but the disease seemed to fix itself upon the parietes of the abdomen, which became heavy, appearing to the patient as if there was a weight upon it when he was lying down; and this symptom, as well as the feebleness of the legs, gradually augmented, at the same time that the pain in the dorsal region persisted.

In this condition the patient entered the Hôtel Dieu, when the following symptoms were observed: Pain in the dorsal region, which was increased by pressure, commencing at the third dorsal and extending to about the first lumbar vertebra; incomplete paraplegia, the sensibility of the trunk to the base of the chest and of the inferior members being perceptibly diminished; partial retention of both the urine and fæces; pains in the abdomen, occasionally lancinating and darting from one point to another. The abdominal parietes appear to the patient constantly on the stretch and as if weighed down by an insupportable load, a sensation which exists equally on the lower part of the thorax, giving rise to an embarrassment in the respiration appreciable only by the patient. He had not had a passage from his bowels in five days. A purgative draught was administered, which produced an abundant discharge. Appetite good; no fever.

The next day two issues were made with the Vienna paste upon the sides of the spine corresponding to the second dorsal vertebra. The following days the symptoms already mentioned made rapid progress, and on the 20th of February the paraplegia was complete, the sensibility of the teguments of the inferior members and trunk to a level with the ribs being entirely abolished. The five superior ribs acted normally, and the diaphragm elevated the abdominal muscles, but it was quite evident that the seven inferior ribs remained stationary during respiration, a symptom which was manifest to the eye and hand applied to the chest. The retention of the urine has become complete; the same is the case with the fæces. Soon after this period an enormous eschar formed on the sacrum and buttocks, and black spots appeared on the toes and on the surface of the internal malleolus.

In the first days of March an erysipelas, which assumed the form of *erysipelas ambulans*, attacked the face and afterwards extended to the arms and upper part of the chest, accompanied by a well marked febrile movement, and continued till the death of the patient.

The patient had repeated chills during the whole of the

18th, and on the 19th he was troubled by a slight but very frequent cough, followed by expectoration of a frothy serum. His countenance now became notably changed; the chills were repeated during the following days, and death ensued on the night of the 23d.

*Autopsy thirty-six hours after death.*—Marasmus extreme, especially in the inferior extremities. The sacrum and buttocks presented a vast wound resulting from the fall of the eschar previously mentioned. The glutei muscles were partly denuded, and partly covered by dark looking flaps of mortified cellular tissue. The slight projection at the second dorsal vertebra observed during life was still to be seen. The spine being opened as carefully as possible, as soon as the spinous processes and posterior portion of the vertebræ were removed, a mass of hydatids was perceived on a level with the second and third dorsal vertebræ, and presented the following disposition: they were enclosed in a cyst about the volume of a man's fist, of an irregular form, longer transversely than vertically; this cyst had elevated the laminæ of the vertebræ, principally those of the right side, which were very thin and seemed in some points even entirely destroyed. Unfortunately the destruction occasioned by the saw prevented our ascertaining the exact state of these parts. The cyst, after having separated or destroyed the laminæ of the second and third dorsal vertebræ, was prolonged underneath the vertebral muscles and for a short distance under the rhomboid muscle. On the left the cyst had only elevated the vertebral laminæ, but it was not prolonged beneath the muscles. Its anterior wall was applied immediately upon the dura mater, which was unaffected. The cord, although compressed, presented no alteration save a slight sanguineous congestion, both under and about the compressed point. The hydatids were of different sizes, some being as large as a cherry, while others were not larger than a pea. The largest were empty, their parietes torn, opaque and elastic, resembling a thin piece of caoutchouc. The smaller were transparent, and filled with a slightly opaline liquid, which, as well as the hydatid cysts, was examined with the microscope, though without detecting the presence of any entozoa. Some traces of inflammation of the mucous membrane of the bronchii, a little sero-sanguinolent engorgement of the pulmonary parenchyma, and a slight effusion in the left pleura, were all that was noticeable in the other portions of the body.

*The action of strychnine on the bladder.*—M. Trousseau, in his *Traité de Thérapeutique*, speaking of strychnine, says: "We have not seen any secretion rendered more active by the nux vomica if it be not the urine, and here not only is the secretion more abundant, but the excretion is both more frequent and more energetic, to such a degree that some patients are obliged to urinate every hour." Trousseau is one of the few writers on medicine who have indicated this predilection, so to speak, of strychnine for the urinary apparatus. Several cases have occurred recently in the wards of M. Vigla, at the Hôtel Dieu, which, while they tend to confirm this opinion, suggest some reflections of the highest practical importance.

The subject of the first case was a man aged forty years, who had been taken five months before, without any known cause, with lassitude and feebleness in the legs, which phenomena gradually became more intense, and ended by constituting a true paralysis of the inferior extremities. He was admitted into the ward, where he was treated at first by revulsions upon the digestive tube, then by strychnine. M. Vigla is accustomed to commence with strychnine in the dose of one-fifth of a grain per diem, given in a gum julep of four ounces. He prefers this mode of administering it both to the endermic method and the form of pills. He has remarked that the effects of the medicine are more rapid and complete when it has been dissolved before reaching the stomach, since this organ thus effects the absorption much more promptly than when both its solution and absorption are required at the same time.

The first effect produced in this case was a more abundant secretion of urine, then frequent desire to micturate, during which act there was slight scalding; subsequently there were twitchings and pinchings in the legs, and a very marked return of mobility, so that the patient was able to walk without much difficulty. The augmented activity of the bladder, the more remarkable as there existed at the time the patient entered the hospital a commencing paralysis of this organ, continued only for a few days, and has diminished in the ratio with which the strychnic phenomena have manifested themselves in the muscles of the extremities.

The second case relates to a man who has been sick for six months. The affection commenced by constipation, difficulty in defecation, sluggishness of the bladder, pains in the back and legs, the latter growing so weak that on his entry, about four months ago, he was totally unable to walk. The treatment that he had undergone before coming to the hospital consisted in venesection, hip baths, ptisane of cherry stones, wine of cinchona and gentian. When examined for the first time, he had almost complete retention of urine resulting from distension of the muscular fibres of the bladder, which were deprived of their elasticity. Belladonna was first prescribed, then strychnine in the same dose and manner as in the case just related. Here, equally as in the preceding example, the first symptoms produced were manifested in the bladder—frequent disposition to urinate, accompanied by scalding during micturition; convulsive twitchings in the muscles of the legs and thighs. At this period (April) the strength has increased, and the patient has commenced walking with considerable facility, although he still throws one of his legs slightly to one side.

The last case is that of a man who, in September last, was attacked with a myelitis, which became chronic. He entered the hospital on the 20th of February, at which time it was wholly impossible for him to move his legs. He was put upon strychnine, and, as in the two others, he experienced tremblings and twitchings in the legs, and even pretty severe pains; a little increase of activity in the secretion as well as excretion of urine. A varioloid, so light that it did not even suppurate, supervened, and the strychnine was obliged to be suspended. But, singular to relate, under the influence of the varioloid, at the end of seven or eight days, the paralysis seemed to be modified, and now the subject, lying upon his bed, can move his foot from the horizontal plane which it has occupied, and lift it to some height. The retention of urine has ceased, and there is no longer any difficulty in its expulsion.

Before proceeding to give some other instances of an analogous character, I may submit, that should ulterior and more extensive experiments with strychnine demonstrate that it has an almost specific action upon the muscular fibres of the bladder, we may hope to derive very great advantages from its employment in certain paralyses of this organ, whether they be idiopathic, the result of some mechanical cause, in which case strychnine is the principal element of the treatment; or symptomatic of some other affection, when it would constitute a useful adjuvant to stimulate the inert viscus, while appropriate means were being directed against the causes of the affection.

M. Mauricet has published in the *Archives de Médicine* (te. xiii, page 403) a short history relating to this subject, which, being unusually striking, I translate entire: The two sons of Mr. R., he says, the one 13, the other 14 years of age, both of lymphatic constitution, had labored since their birth under nocturnal incontinence of urine. I prescribed the alcoholic extr. of nux vomica in doses of ½ gr. morning and evening. Three days elapsed; the incontinence had disappeared and was not again seen during the use of the remedy. At the end of fifteen days, the nux vomica was discontinued; relapse. Consulted again, I made the same prescription; the incontinence again disappeared. The treatment was interrupted; another relapse. Finally, having taken the extract for the third time, and having continued its use during a month, the two patients were completely cured of their disagreeable affection.

M. Mauricet, after observing concerning these facts that they require to be substantiated by new experiments, adds— "Nevertheless, in considering that the incontinence of urine has always disappeared under the influence of the strychnia, and that it manifested itself anew at the cessation of the remdy, have we not ground for believing that the nux vomica contributed powerfully to the cure ?"

One more case, and I dismiss the subject: M. Trousseau employed strychnine with the most perfect success in a woman who, in consequence of a fall from a considerable height, had been first paraplegic, and afterwards merely affected with a paralysis of the bladder. The latter affection yielded most promptly to the strychnine.

Since M. Velpeau has not played a conspicuous part in my letters for some time past—owing more than anything else to my having devoted the months of January, February and March especially to the study of medicine—I have concluded to report one of the lectures which he delivered a week or two since, and which I believe will be found to possess all the interest that usually attaches to the clinics of this eminent professor. It is as follows:

*Cancer of the breast.*—A peasant about fifty years old has returned to our service for a cancer of the breast. I say returned, for she had presented herself to us when the tumor was circumscribed, its limits clearly definable, its mobility evident—in a word, when it offered conditions favorable to the operation. But the tumor was the seat of no pain; the patient could not comprehend how a tumor which gave her no trouble was a fit subject for the knife, and, refusing the only efficacious remedy that we could propose, returned to the country. Today she asks for what at that time she could not be prevailed upon to accept; today the tumor is ulcerated, adherent, and extends perhaps to the ribs. This is a trouble that you will often meet with in practice. You will see the operation rejected at the moment when it is opportune, and solicited at a later period, when the progress of the disease has rendered it almost impossible, or at best of doubtful success. And this is particularly the case when the cancer causes no pain, especially as women find physicians who give them counsel more in accordance with their taste in altogether discountenancing the operation or in postponing its application. This order of physicians may be subdivided into numerous genera. The first are charlatans, whose only end is to inspire the patients with a false security, the consequences of which it is needless to allude to. There are others, and these may perhaps be conscientious, who believe in the medical cure of cancer, at least in certain cases; these essay internal medication before resorting, if it should become necessary, to the removal of the tumor. It is not true that medicine ever made the smallest cancer disappear, and these pretended cures arise from an error in diagnosis. In good practice, he alone attacks cancer by internal remedies who is assured that the nature of the disease is not malignant. And should he have to deal with confirmed cancer, he is in a dangerous path; he loses precious time in dissipating or diminishing the engorgement of the tissues which surround the tumor, while he exercises not the slightest influence on the final result. This method, then, possesses not a single advantage, while it has many attendant evils.

In the first place, that cancer is often primitively a local affection I have not the slightest doubt, although this is a point which, as you know, has been warmly contested. In temporizing, then, or leaving the disease to become general, the cancerous cellule, if it really exist, is transported first into the circulation, afterwards into the other organs, and infection is the result. Granting, even, that the cancerous principle preexisted in the economy, and that the tumor is but a manifestation of it, in removing this tumor, if you do not destroy the principle, you at least destroy one of its effects, without increasing in any degree the activity of the cancerous diathesis.

On the other hand, as a wound the operation offers no danger if it be made in time, a little while after the appearance of the morbid product—when, for example, its volume does not exceed that of a filbert. In this case you relieve the patient by a small incision scarcely followed by reaction. I should advise you, and this is my practice, to operate as soon as the cancerous character is evident. To wait till the tumor ulcerates and extends, or even until the ganglia become affected, is to compromise the life of the patient as well as the character of surgery. When the ganglia are only *engorged*, the result is already uncertain, and if you would operate under such circumstances now, you will not do it when you become old. Young practitioners attribute this to the coldness of age, and, full of confidence in the powers of the art the duties of which they are just entering upon, they mistake for timidity what is but the fruit of experience. And after a first and even a second failure, they still repeat their efforts; but finally correcting themselves, they in their turn become old, and no more subject their patients to useless torture.

In the case which occupies us, the tumor is ulcerated, adherent, comprehending perhaps the the ribs, and the ganglia in the axilla are enlarged. This lymphatic engorgement, which is nearly always cancerous, would deter me from any operation if the patient, seeing but too clearly the fate that awaits her, had not persuaded me by her repeated and anxious entreaties to give her, uncertain as it is, the sole chance that remains. The condition of the part, and especially its size and depth, forbids removal by the bistoury; caustics are scarcely of easier application, but they disturb the economy less, occasion no fever, and although more painful, are less alarming to the patient. To what caustic should we give the preference—to the paste of chloride of zinc, or that of Vienna, or that of frére Côme? The latter possesses a particular danger, belonging to the poisonous nature of arsenic, which constitutes its base; and although these dangers have been exaggerated, still they are not the less real, as there are instances to prove. And here the size of the absorbing surface augments it in a fearful degree. Besides, this paste produces great pain and high inflammation. That of Vienna produces a sanguineous discharge which fuses it, and its action is too superficial. That of the chloride of zinc attacks only fungous tissues or those deprived of their epidermis; you may hold it a year in your hand without feeling it, but the moment you remove the epidermis by a blister, it will take effect and burn you violently—a property as true as it is strange. It would be necessary here to denude a part of the tumor, and this initial step is very embarassing; and let me add that this paste causes cruel suffering during the whole time of its application.

The *black caustic*, composed of sulphuric acid and saffron, without any precise formula, but so as to form a homogeneous paste, appears to me to possess incontestable superiority over all the others. It destroys every surface with which it comes in contact; it occasions no sanguineous discharge even when the skin is ulcerated and fungous; it occasions very little pain; the tissues attacked become dry, and suppuration arrives only with the eliminatory inflammation at the end of fifteen days; and during this time, without any dressing, without any care, the patient may forget his eschar. Added to all the rest, the retraction of the eschar limits the extent of the cicatrix. It is true that its application is somewhat difficult; it adheres more to the spatula than to the tissues. As it burns all, the diachylon cannot circumscribe it; it is not well applied except on a horizontal surface, and it is, moreover, liable to become fused. But these defects, which I am far from endeavoring to conceal, by no means counterbalance its good qualities, and I repeat that the black caustic is, in my opinion, preferable to all the others. I proceed to attack successively the various points of the tumor by partial applications.

In the latter part of March Prof. Velpeau delivered another lecture on the subject of cancer of the breast, which consisted in substance of the following:

The patient on whom we are about to operate is a woman forty years of age, of a dry and nervous temperament. She is affected with a tumor of the right breast, which dates, according to her account, only about four months back, and was caused by a blow with a key. I saw the patient a month after the accident to which she attributes the development of the tumor, and it was apparently about the same volume then as it is now. The nature of this morbid production does not allow of our believing in so rapid a growth. And here I may remark that this is a shoal with which you will often meet in the world, and which you must learn to avoid. Persons will tell you that a considerable tumor has appeared within a few months, and in some cases even within a few days, when in reality it has existed for years. A great many tumors commence and attain considerable size without having by any pain, by any embarrassment, awakened the attention of the patient. It is especially in the breast, and in those that are voluminous, that this latent state may be developed. The tumor there is lost in the tissue which envelops it, and if it does not deform the organ, it escapes the attention of the patient who does not examine the part. And when from a blow, or violence, or any suffering whatever, this examination is made and the tumor is discovered, by an innate tendency to search for the causes of things, the woman sees in the blow of a key, in the pressure of the whalebone of her corset, little mishaps that are so slight as scarcely to be remembered, the cause of her malady. At other times, it is by chance that the disease is perceived. In the present case the breast was neither voluminous nor deformed, but the deception had another source. Instead of a circumscribed tumor in the mamma, it is the entire gland that is invaded. The breast has not changed either in form or size, but in its consistence and nature; it has hardened and degenerated, and as this transformation has been accomplished without pain, it remained for a long time unknown. We cannot accept the testimony of the patient, for it is scarcely possible that a sanguineous tumor could so promptly have acquired such volume and hardness; and it is evidently of a scirrhous or encephaloid character, the uniform manner in which the entire gland is affected, the inequalities and the consistence of the tumor, removing all doubt in this respect. And what unfortunately still further confirms our diagnosis is the engorgement of the axillary ganglia of the corresponding side, and the history of the family of the patient, her mother having died from cancer of the uterus, and her sister from cancer of the breast.

Whether scirrhous or crude encephaloid, we undoubtedly have to do with a cancer, which we may safely say commenced not less than eighteen months ago. This cancer, of hereditary origin, and complicated with engorgement in the ganglia of the axilla, does not afford sufficient prospect of success to propose an operation to the patient. So far from it, indeed, I have only consented to attempt an operation after much entreaty. But this woman having seen her sister die from a mammary cancer which was not operated upon, she has returned so many times to the charge that I am at length overcome. I have been overcome not only by her entreaties, but by a prospect of the danger which I feel menaces her, left to herself—the inflammation of the cancer, the excrescences which are reproduced, the repeated hemorrhages, the incessant sufferings, and, at the end of all that, a cruel death—this more than all else has made me yield. Besides, a relapse does not always occur, and may be very distant.

The disease comprehending all the breast, it is necessary to amputate the whole of the organ. As the tumefaction of the ganglia may be owing to a simple inflammation, we will preserve them for the present, extirpating them at a future period if the engorgement of which they are the seat shows a malignant character. The patient strongly wishes to be etherized, and as there is no reason why she should not be, we will administer the ether.

The patient at first did not breathe the ether well, but she soon commenced inspiring more fully, and at the end of five minutes became insensible. During the operation, which lasted a minute and a half, there was no sign of pain. There did not in fact seem to be any suffering, nor even a dream of it—nothing but insensibility attended by a perfect calm.

Eight days after the operation, Prof. Velpeau returned to the case thus:

The day after the operation, very severe pains, though without fever, declared themselves about the left breast, on the side opposite to the one which had been removed. The woman was not alarmed, because, she said, she was subject to these nervous attacks. The third day this pain had changed its seat to the epigastrium, and had become violent and distressing. There was nothing noticeable on the part of the wound or elsewhere, except an insignificant erysipelas on the shoulder. I prescribed calmants.

On the fourth day the pains redoubled themselves, extending throughout the abdomen and reaching the base of the chest; smallness of the pulse, attended with violet hue of the face, and threatened asphyxia; followed on the fifth day by death. This event, which was as unexpected and sudden as it was violent, seemed to have its origin in what the ancients called *phrenitis*, that is to say, inflammation of the serous surfaces of the diaphragm; and we shall without doubt find, to-morrow, a peritonitis, perhaps a pleurisy, and possibly also a pericarditis.

The day after the autopsy, the professor expressed himself in these terms:

The idea, the fear that etherization plays some part in the development of such accidents, made it our duty to examine most scrupulously the lungs, although there was scarcely any embarrassment in their functions, and then only in the last moments of life. But the state of the bronchii and of the pulmonary tissue offers. as you see, nothing abnormal. There is only at the base of the two viscera a very ordinary alteration, and, so to speak, of hypostatic engorgement, an engorgement which is not very decided and without induration. You will remark the traces of violent inflammation upon the two diaphragmatic surfaces—of pus and false membranes in the pleura and in the peritoneum. The plastic layers are very thick, particularly on the spleen and liver. This is a result that I have witnessed more than once before the ether was heard of. It is not very rare to see intercurrent phlegmasias, especially pleurisy, manifest themselves after amputation of the breast; and I saw a fatal peritonitis follow the same operation in bed No. 31 of the same ward, seven years ago. The patient died the third day, of the peritonitis.

Men are no more exempt from these complications than women; and what is remarkable, these accidents, instead of waiting until the day after the operation, sometimes appear in the evening of the day on which it is performed. I shall always remember a vigorous young man who came to La Pitié

to be operated upon for a lachrymal fistula, and was carried off by an acute peritonitis. Here we have had a delicate, nervous and susceptible woman operated upon in a badly heated room, having had to traverse cold passages both coming to and going from the amphitheatre. These are the facts: I leave you to judge respecting matters of which you were eyewitnesses.—The tumor was of a fatty texture and extremely hard.

*A case of sarcocele.*—The day subsequent to this, Professor Velpeau operated for sarcocele, and I have thought it worth while to connect two affections so analogous:

The patient is thirty-nine years old, affected with a tumor of the testis. This tumor, which is not of very recent date, is of the volume of my fist, ovoid, regular, indurated, and of the form of a hydrocele, though on examining it with care we soon see that there exists no transparency and no trace of fluctuation, and by pressure we detect a solid elastic body. The tumor is of considerable weight. But you must bear in mind, remarked the professor, that in hydroceles of long standing, the scrotum being thickened, you may observe the greater part of these characters. An explorative puncture gave rise to the discharge of a little blood, and I was able to feel that the point of the trocar was implanted in a body of a concrete nature.

Concrete tumors of the scrotum all bore once the generic name of *sarcocele*, but gradually the application of the word was restricted; scrofulous and fibrous tumors and hypertrophies of the gland were one by one excluded, until today the name of sarcocele is applied only to cancer of the testicle. Hypertrophy of the testicle does not acquire the volume of the tumor that we have here; it attacks the two testicles, does not afford so great a density, has a certain softness, and is, moreover, rare in our country. I have observed many cases of it among Brazilians.

The tuberculous testicle rarely possesses this volume; it is unequal, embossed with certain projections, which are soft, painful, and at times contain pus.

Fibrous tumors are rare; nevertheless they are occasionally met with; they have almost exactly the volume, consistence and march of this. There is no reason except their rareness why we should not suspect one here, though as cancer is much more common, it is natural to apprehend that this is one.

Cancer of the testicle is almost always of complex nature: we find it encephaloid, scirrhous, or colloid. We are not able, then, *a priori*, to determine the nature of the tissue of the tumor; nevertheless everything induces the belief that this is formed of encephaloid tissue. Whatever it may be, the remedy is the same, namely, castration. For the prognosis, it is to be desired that the tumor were of a fibrous nature, for then we should be sure to have no return of the affection.

The patient inspired the ether; and as he feared the pain of the operation, was told that this would be deferred till the next day. At first he respired the ether rather imperfectly, but presently seized the apparatus as you have so often seen persons do when breathing the nitrous oxide, and in the space of three minutes he was pricked without seeming to feel it. At the first stroke of the bistoury he cried out, "Let me alone," and then became a little disturbed. The operation was continued and the tumor dissected; the patient spat, and said, "No danger, no danger." The cord was ligatured *en masse*, and the patient did not utter a single cry or make the slightest motion. During the dressing, gradually returning to himself, he said—"You may operate upon me now; I am sure I shall feel nothing."

*Excision of the tonsils.*—A child being placed upon the table whose tonsils it was necessary to remove, Prof. Velpeau remarked:

I consider the *tonsillitome* preferable to the bistoury, especially at this age. I do not pretend that it should be used in all cases. It is necessary that the tonsil be globular and isolated; if it has a large base, the bistoury will be more convenient, as with that instrument you are able to dissect a little before excising it. Another difficulty presented by the *tonsillitome* is that if, the tonsil being once engaged, the spit should tear it, you only break the gland. In order to avoid this accident, you must, in depressing the handle of the tonsillitome, bring the amygdala entirely within the ring before driving in the spit. It is because this maneuver is either not understood or neglected, that the operation is so often imperfectly performed. As the volume of hypertrophied amygdalæ is exceedingly variable, you should provide yourselves with at least two tonsillitomes, one for children and the other for adults. If you should, however, have but one, and the ring of this, as in some instances it is, be too large, by placing the anterior segment upon the gland—which, thus retained, will not escape when the blade arrives—you will usually succeed. It has been said that the tonsillitome was invented for the awkward, but this should not be a reproach, for you see there is a proper way to use it. It possesses, moreover. the advantage of rendering the operation more prompt, more certain, and at the same time less painful.

Paris, April 30, 1847.

